# Point-of-Care Troponin T Testing in the Management of Patients with Chest Pain in the Swedish Primary Care

**DOI:** 10.1155/2013/532093

**Published:** 2013-01-10

**Authors:** Staffan Nilsson, Per O. Andersson, Lars Borgquist, Ewa Grodzinsky, Magnus Janzon, Magnus Kvick, Eva Landberg, Håkan Nilsson, Jan-Erik Karlsson

**Affiliations:** ^1^Primary Care, Department of Medical and Health Sciences, Faculty of Health Sciences, Linköping University, East County Primary Health Care, County Council of Östergötland, 581 83 Linköping, Sweden; ^2^Central County Primary Health Care, County Council of Östergötland, 581 85 Linköping, Sweden; ^3^Primary Care, Department of Medical and Health Sciences, Faculty of Health Sciences, Linköping University, 581 83 Linköping, Sweden; ^4^Division of Biomedical Laboratory Science, Department of Medical and Health Sciences, Faculty of Health Sciences, Linköping University, Department of R&D Unit in Local Health Care, County Council of Östergötland, 581 85 Linköping, Sweden; ^5^Division of Cardiovascular Medicine, Department of Medical and Health Sciences, Faculty of Health Sciences, Linköping University, 581 85 Linköping, Sweden; ^6^Department of Cardiology UHL, County Council of Östergötland, 581 85 Linköping, Sweden; ^7^East County Primary Health Care, County Council of Östergötland, 601 82 Norrköping, Sweden; ^8^Division of Clinical Chemistry, Department of Clinical and Experimental Medicine, Linköping University, County Council of Östergötland, 581 83 Linköping, Sweden; ^9^Division of Cardiology, Department of Internal Medicine, County Hospital Ryhov, 551 85 Jönköping, Sweden

## Abstract

*Objective*. To investigate the diagnostic accuracy and clinical benefit of point-of-care Troponin T testing (POCT-TnT) in the management of patients with chest pain. *Design*. Observational, prospective, cross-sectional study with followup. *Setting*. Three primary health care (PHC) centres using POCT-TnT and four PHC centres not using POCT-TnT in the southeast of Sweden. *Patients*. All patients ≥35 years old, contacting one of the primary health care centres for chest pain, dyspnoea on exertion, unexplained weakness, and/or fatigue with no other probable cause than cardiac, were included. Symptoms should have commenced or worsened during the last seven days. *Main Outcome Measures*. Emergency referrals, patients with acute myocardial infarctions (AMI), or unstable angina (UA) within 30 days of study enrolment. *Results*. 25% of the patients from PHC centres with POCT-TnT and 43% from PHC centres without POCT-TnT were emergently referred by the GP (*P* = 0.011 ). Seven patients (5.5%) from PHC centres with POCT-TnT and six (8.8%) from PHC centres without POCT-TnT were diagnosed as AMI or UA (*P* = 0.369). Two patients with AMI or UA from PHC centres with POCT-TnT were judged as missed cases in primary health care. *Conclusion*. The use of POCT-TnT may reduce emergency referrals but probably at the cost of an increased risk to miss patients with AMI or UA.

## 1. Introduction

Chest pain is a frequent complaint in primary health care and a daily diagnostic challenge to the general practitioner (GP). The outcome of GPs' diagnostic capability has been investigated and clinical decision rules have been suggested based on history, symptoms, signs, and electrocardiogram findings [1–5]. Elevation of cardiac-specific troponins, for example, Troponin T, is one of the cornerstones in diagnosing acute myocardial infarction (AMI) in hospital care [6]. The additional value of point-of-care Troponin T testing (POCT-TnT) in primary health care has not been fully evaluated [7–9]. On one hand, support from POCT-TnT may reduce referrals to the emergency room, but on the other hand a very recent AMI or unstable angina (UA) may be overlooked if the GPs rely too much on a laboratory finding.

The aim of this study was to investigate the diagnostic accuracy and clinical benefit of POCT-TnT in the management of patients with chest pain in a primary health care setting.

## 2. Material and Methods

### 2.1. Study Design

The study was performed between May 2009 and January 2011 in the county of Östergötland, situated in the southeast of Sweden. It was an observational, prospective, crosssectional study with followup. Patients consulting their GP for chest pain in three primary health care (PHC) centres using POCT-TnT were compared to patients consulting for chest pain in four PHC centres not using POCT-TnT. POCT-TnT was already in routine use in the three PHC centres. No specific training on the properties of POCT-TnT was done in preparation for the study. In October 2009, the number of listed patients older than 35 years ranged from 3698 to 4123 in the PHC centres using POCT-TnT and from 3366 to 7074 in the PHC centres not using POCT. As the recruitment of patients from PHC centres not using POCT-TnT was limited despite repeated contacts and encouragement from the investigators, two of the four PHC centres without PCTT were recruited in June and July 2010, respectively.

### 2.2. Point-of-Care Troponin T Testing (POCT-TnT)

Blood was collected by venipuncture in vacuum tubes containing separating gel and lithium heparin (4 ml, Greiner Bio-One, Frickenhausen, Germany). Instantly after sampling, Troponin T was measured in whole blood on the point of care test (POCT) instrument Cobas h232 (Roche Diagnostics, Mannheim, Germany). The time required for measurement was 14 minutes as a maximum. The instrument and each lot of test cassettes were tested regularly by using control material with established values of Troponin T. The detection limit was 0.03 *μ*g/L and all values >0.03 *μ*g/L were regarded as positive according to recommendations from Roche. This limit was in accordance with the decision limit used for Elecsys Troponin T, third-generation (Roche) based on a coefficient of variation of less than 10%. The 99th percentile for a healthy population is ≤0.01 *μ*g/L [10]. In the interval between 0.03 and 0.1 *μ*g/L results were recorded as “0.03–0.1 *μ*g/L.” Quantitative values were generated between 0.1 and 2 *μ*g/L.

### 2.3. Data Collection

**Figure 1:**
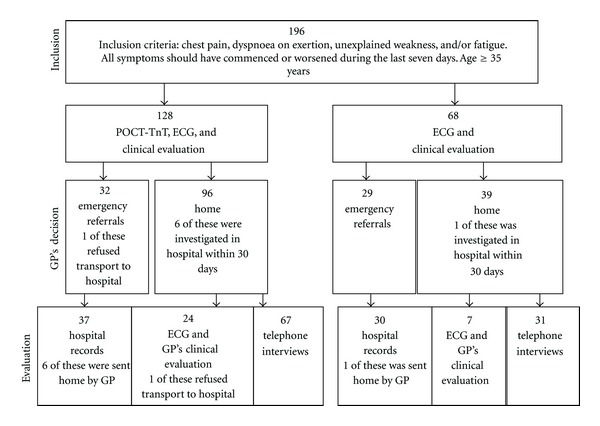
Patient flow in primary health care centres (PHC centres) with and PHC centres without point-of-care Troponin T testing (POCT-TnT). Decisions by general practitioner (GP) and methods of end point evaluation are shown. Exclusion criteria: severely affected patients. Other probable cause of chest pain than cardiac, according to a nurse's telephone assessment, for example, costal fracture or gastrooesophageal reflux.

In all seven PHC centres all patients were, according to normal routines, given an appointment with their GP after calling the PHC centre and talking to a nurse, who included all eligible patients according to inclusion criteria (Figure 1). In addition, the GPs were asked to include eligible patients in conjunction with consultations.

Management of the patients was noted by the GPs on the case report form (CRF) developed for the study.

After three to five weeks all patients were contacted for a structured telephone interview by a research nurse. The interviews included questions about any further consultations to the emergency department (ED) or hospitalisations for chest pain, dyspnoea, fatigue, or any other heart-related symptoms. Patients fully evaluated in hospital, whether AMI/UA or not, during study time were not further investigated. All other patients reporting discomfort in the chest on exertion or avoiding strenuous activities were scheduled for an appointment with one of the GPs in the study (Figure 1). Current symptoms of angina pectoris were graded according to Canadian Cardiovascular Society Classification I–IV and explored whether new, unchanged, impaired, or improved compared to symptoms at study inclusion [11]. An electrocardiogram (ECG) was registered. Aiming to find any possible further visits to the ED or hospitalisations within 30 days after study inclusion, the computerised medical record system was searched. To ensure data quality, a 100% source data verification of the CRF was performed for 20 randomly selected patients by an external monitor who also performed a general overview of all data in the database.

### 2.4. Patients Assessed in the Emergency Department or Hospitalised within Thirty Days after Inclusion

Hospital medical records for all patients evaluated at the EDs or hospitalised for chest pain or any heart-related symptoms within 30 days were reviewed by one of two cardiologists who were uninformed about the aim of the study. The reviews were performed using a systematic protocol aiming to verify or rule out AMI or UA. In cases of uncertainty the two cardiologists each made independent reviews and thereafter conferred to reach consensus. All seven participating PHC centres and the three hospitals' clinics used the same computerised medical record system. In order to minimise bias, all current primary health care and hospital records were printed on paper and made anonymous to the reviewers.

For those patients sent home after the GP's assessment, but admitted to the ED or hospitalised within 30 days, the primary health care medical records, ECG, and laboratory findings were reviewed. This review was made, using a systematic protocol, by one GP and one cardiologist independently. Both were uninformed about the aim of the study. After this independent review, the GP and the cardiologist were asked to confer to reach consensus. The aim of this review was to decide if the patient should be considered as a missed case of AMI or UA in primary health care [12].

### 2.5. Statistical Analysis

The Pearson Chi-square test and the Fisher's exact test were used for nominal and the independent samples *t*-test for continuous variables. A *P* value below 0.05 was considered significant.

### 2.6. Ethics

The study was approved by the Regional Ethical Review Board in Linköping, Sweden, Dnr M101-09, T98-09, and Dnr 2010/211-32. Written informed consent was obtained from all patients before study enrolment.

## 3. Results

A total of 196 patients were included, 128 in PHC centres with POCT-TnT and 68 in PHC centres without POCT-TnT. There were no significant differences between the two groups concerning age, gender, cardiovascular risk factors, or history of cardiovascular disease (Table 1). Fewer patients from PHC centres with POCT-TnT (*n* = 32, 25%) were emergently referred to hospital than from centres without POCT-TnT (*n* = 29, 43%), (*P* = 0.011), (Table 2). However, a follow-up visit to the GP was scheduled more often in PHC centres with POCT-TnT than in centres without POCT-TnT (*P* = 0.013), Table 2.

Seven patients (5.5%) from PHC centres with POCT-TnT were diagnosed with AMI or UA compared to six patients (8.8%) from centres without POCT-TnT (*P* = 0.369), (Table 3). In all these 13 cases, the time between onset of symptoms to examination by the GPs in the PHC centres was at least 10 hours. Five of the 128 patients had a positive TnT value (>0.03 *μ*g/L). In the remaining 123 patients, the result was negative. The median time to the GPs' followup of the 24 and 7 patients in each group was 37 and 35 days, respectively (Figure 1). There was no report of signs of AMI after study inclusion according to ECG findings. No patient had UA, but seven and four, respectively, were diagnosed having angina pectoris. One of the patients from PHC centres without POCT-TnT reported angina pectoris with worsening symptoms which, however, according to review of the medical records was not a case of UA.

Within 30 days, three of the patients sent home by GPs at the PHC centres with POCT-TnT were diagnosed as AMI or UA. Two of these, one AMI and one UA, were judged as missed cases in primary health care (Table 3). The third case, not considered to be a missed case, was an 84-year-old women diagnosed with AMI 26 days after the visit to the GP. There was full agreement between the GP and the cardiologist considering this judgement.

The sensitivity of the GPs' decision for emergency referral in relation to a later confirmed diagnosis of AMI was 67% among patients assessed with POCT-TnT and 100% among those assessed without POCT-TnT. Corresponding figures for AMI and UA were 71% and 100%, respectively (Table 4). The sensitivity of POCT-TnT to find a patient with AMI was 67% (95% CI, 9.4–99.2) and to find an AMI or UA 29% (95% CI 3.7–71.0). The specificity of POCT-TnT was 98% (Table 5).

## 4. Discussion

GPs in PHC centres with POCT-TnT more often refrained from emergency referral of chest pain patients than GPs in PHC centres without POCT-TnT. However, there were two cases of missed diagnosis of AMI or UA in PHC centres with POCT-TnT and none in PHC centres without POCT-TnT. More patients from PHC centres with POCT-TnT were booked for a follow-up visit to their GP.

A major strength of the study was the prospective design and thorough followup regarding possible diagnoses of AMI or UA. The study can be regarded as a cross-sectional diagnostic study with delayed reference standard [13]. The reference standards were performed and interpreted using standardised criteria. Hence, an expert panel of two independent cardiologists used criteria and decision rules for a clinical agreement and assigned a final diagnosis to each patient, based on the available clinical information. A limitation of the study is the slight possibility of silent AMI among those who reported no symptoms and were not hospitalized within 30 days. However, we assessed this risk as very low and therefore omitted these patients for followup. A second limitation of the study was that the PHC centres were not randomised (due to practical reasons) whether to use POCT-TnT or not. However, the study could be regarded as a quasiexperimental study using the Troponin measurement as the intervention and the AMI/UA diagnoses as the outcome. The quasiexperimental design reduces threats to external validity as the natural environment does not suffer the same problems of artificiality as compared to a controlled laboratory setting. Hence, the design might facilitate followup and application on other primary care centres. A third limitation was that recruitment of patients in PHC centres with POCT-TnT was higher compared to centres without POCT-TnT. We attributed this to a difference in study awareness; that is, the possibility to analyse Troponin T instantly made GPs and nurses more aware of the study. In PHC centres without POCT-TnT there were only the study protocols to remind of the study. We aimed to compensate for this imbalance through repeated reminders through telephone calls and personal visits.

To our knowledge, there are no studies where clinical sensitivity and specificity have been thoroughly investigated for the POCT-TnT on Cobas h232. Bertsch et al. investigated the correlation of POCT-TnT to the laboratory TnT method in the measuring range (0.1–2.0 *μ*g/L) and concluded that there was a good analytical agreement [14]. However, no evaluation of clinical sensitivity was done, which would have involved a comparison of TnT values below 0.1 *μ*g/L, that is, the most interesting level in primary care.

The combined prevalence of AMI and UA was 5.5% and 8.8% in each study group. In comparison, the prevalence of AMI and UA is about 22–36% of the chest pain population at the ED [15, 16]. In general, the probability of AMI and UA in our study population was low as severely affected patients were excluded. Including those patients would probably have enhanced sensitivity figures for GP's decision in the PHC centres with POCT-TnT (Table 4) and for POCT-TnT for AMI (Table 5(a)). It must be emphasised that the aim of the study was to investigate the additional value of POCT-TnT to rule out AMI and UA in a low-risk population. The risk of a false negative POCT-TnT due to very short duration of symptoms must be assessed as minimal since cardiac Troponin T begins to rise within four hours after the onset of myocardial injury and the time from onset of symptoms to taking the blood sample was at least ten hours [17]. Cardiac Troponin T can remain increased for up to 14 days after myocardial injury thus covering the seven-day time limit defined in the inclusion criteria [6, 17].

Our results must be interpreted with caution given the small number of AMI and UA, and since it was not a randomized study. In a study by Planer et al. published in 2005, POCT-TnT was mandatory for all study patients and there was no control group [7]. In their study the sensitivity of the GP's decision combined with the results of POCT-TnT was 100% for AMI, to be compared with our results where it was 67% (Table 4). We chose to analyse the diagnostic accuracy of POCT-TnT for both UA and AMI which may be debatable since an elevation of Troponin T is one of the diagnostic markers for AMI but not for UA. However, in a similar study by Tomonaga et al., the diagnostic accuracy of POCT-TnT for both AMI and UA was analyzed [18]. The clinical presentation of AMI and UA may be identical so the risk of omitting UA based on a negative POCT-TnT is obvious. Referring to the actual decision level for AMI, that is, 0.015 *μ*g/L, together with the kinetic course of Troponin concentration, there is also a risk of omitting an AMI.

Two patients from PHC centres with POCT-TnT were judged as missed cases of AMI or UA, and none from the other group. It is tenable to suggest that the negative POCT-TnT results contributed to these miss managements. In the study by Tomonaga et al., the sensitivity was also found to be higher in the control group compared to the POCT-TnT group, 100 and 90 percent, respectively [18]. Their study had a control group but, in contrast to our study, theirs was cluster randomised. Their Troponin method had a higher detection limit, (0.05 *μ*g/L), as compared to ours, (0.03 *μ*g/L).

A German study not dealing with POCT-TnT found that GPs' diagnosed acute coronary syndromes with the sensitivity of only 50% and that only 41% of these patients were referred immediately to hospital [19]. This is in contrast to our study, where GPs without POCT-TnT did not fail to refer a single patient with AMI or UA. The sensitivity of POCT-TnT to find an AMI was low (67%) and to find an acute coronary syndrome even lower (29%). Despite wide 95% confidence intervals these findings are notable. False positive POCT-TnT was not a problem demonstrated by the high specificity, that is, 98% (Table 5).

### 4.1. Management in PHC Centre

More of the patients were booked for a follow-up visit at PHC centres with POCT-TnT compared to PHC centres without POCT-TnT. Possibly these follow-up visits were planned as an extra check in cases of uncertainty, which is an important method in managing primary care patients with diffuse symptoms. However, one can speculate whether GPs, with the support of a negative POCT-TnT, chose to follow up the patients themselves rather than to make an emergency referral.

## 5. Conclusion

The use of POCT-TnT may reduce emergency referrals but probably at the cost of an increased risk to miss patients with AMI or UA. Swedish physicians at PHC centres do not seem to need the aid of POCT-TnT analysis to improve the chance of finding patients with AMI or UA. An ideal point-of-care cardiac biomarker for use in primary care should have near 100% sensitivity and be able to exclude AMI and possibly also UA with high accuracy. The purpose would mainly be to reduce the number of emergency referrals. This study emphasizes that before introducing new tests for cardiac markers in primary care it is important to evaluate the outcome, preferably by a large enough randomised study.

## Figures and Tables

**Table 1:** Clinical characteristics of chest pain patients managed in primary health care (PHC) centres with and without point-of-care Troponin T testing (POCT-TnT).

	Patients from PHC centres with POCT-TnT *n* = 128	Patients from PHC centres without POCT-TnT *n* = 68	*P* value
Demographics			
Age, years mean ± SD	66 ± 14	65 ± 13	0.670
Male, *n* (%)	71 (56)	42 (62)	0.396
Presenting symptom			
Chest pain, *n* (%)	110 (86)	60 (88)	0.652
Weakness and/or dyspnoea on exertion, no chest pain, *n* (%)	18 (14)	8 (12)	0.652
Risk factors			
Current smokers, *n* (%)	15 (12)	10 (15)	0.787
Diabetes, *n* (%)	20 (16)	5 (7.4)	0.098
Hypertension, *n* (%)	47 (37)	28 (41)	0.541
Hypercholesterolemia, *n* (%)	36 (28)	16 (24)	0.488
Cardiovascular disease			
Angina pectoris, *n* (%)	22 (17)	10 (15)	0.655
Previous AMI, *n* (%)	20 (16)	8 (12)	0.462
Coronary revascularisation, *n* (%)	16 (13)	6 (8.8)	0.438
Stroke, *n* (%)	5 (3.9)	2 (2.9)	1.000
Heart failure, *n* (%)	12 (9.4)	2 (2.9)	0.144
Aortic valve disease, *n* (%)	6 (4.7)	3 (4.4)	1.000
Potential causes of elevation of Troponin T in the absence of overt ischemic heart disease^1^, *n* (%)	3 (2.3)	0 (0)	1.000
ECG			
Sinus rhythm, *n* (%)	114 (89)	62 (91)	0.890
Atrial fibrillation, *n* (%)	12 (9.4)	5 (7.4)	0.890

^1^That is, hypertrophic cardiomyopathies, renal failure, or amyloidosis.

**Table 2:** Management of chest pain patients in primary health care (PHC) centres with and without point-of-care Troponin T testing (POCT-TnT).

	Patients from PHC centres with POCT-TnT *n* = 128^1^	Patients from PHC centres without POCT-TnT *n* = 68^1^	*P* value
Management in PHC centres			
Emergency referral, *n* (%)	32 (25)	29 (43)	0.011
Another visit booked, *n* (%)	18 (14)	2 (3.0)	0.013
Telephone call, *n* (%)	25 (20)	9 (13)	0.276
Back when necessary^2^, *n* (%)	52 (41)	27 (40)	0.083

^1^Information missing for one patient not emergently referred. ^2^No contacts planned by the GP.

**Table 3:** Chest pain patients with acute myocardial infarction (AMI) or unstable angina (UA) from primary health care (PHC) centres with and without point-of-care Troponin T testing (POCT-TnT).

	Patients from PHC centres with POCT-TnT *n* = 128	Patients from PHC centres without POCT-TnT *n* = 68	*P* value
Acute myocardial infarction, *n* (%)	3^1^ (2.3)	5 (7.4)	0.129
Unstable angina, *n* (%)	4^1^ (3.1)	1 (1.5)	0.660

^1^One AMI and one UA judged as missed cases in primary health care.

**Table 4:** Diagnostic accuracy of GPs' decision to refer chest pain patients emergently, with and without the support of point-of-care Troponin T (POCT-TnT).

		Sensitivity		Specificity		PPV		NPV	
		*n*	%	*n*	%	*n*	%	*n*	%
GP's decision with POCT-TnT *n* = 128	AMI^1^	2/3	67	95/125	76	2/32	6,3	95/96	99
	AMI + UA^2^	5/7	71	94/121	78	5/32	16	94/96	98
GP's decision without POCT-TnT *n* = 68	AMI	5/5	100	39/63	62	5/29	17	39/39	100
	AMI + UA	6/6	100	39/62	63	6/29	21	39/39	100

^1^Acute myocardial infarction, ^2^unstable angina.

**Table:** (a)

		“AMI”	“No AMI”		
Point of care	>0.03 *μ*g/L	2	3	5	+PV = 2/5 = 40%
Troponin T	<0.03 *μ*g/L	1	122	123	−PV = 122/123 = 99%

		3	125	128	

Sensitivity 2/3 = 67%, specificity = 122/125 = 98%.

**Table:** (b)

		“AMI + UA”	“No AMI + UA”		
Point of care	>0.03 *μ*g/L	2	3	5	+PV = 2/5 = 40%
Troponin T	<0.03 *μ*g/L	5	118	123	−PV = 118/123 = 96%

		7	121	128	

Sensitivity 2/7 = 29%, specificity = 118/121 = 98%.
